# Post-exposure prophylaxis with sotrovimab for Omicron (B.1.1.529) SARS-CoV-2 variant during the aplastic phase of autologous stem cell transplantation

**DOI:** 10.1186/s13027-022-00454-y

**Published:** 2022-08-03

**Authors:** Gianpaolo Marcacci, Nicola Coppola, Emanuela Madonna, Cristina Becchimanzi, Stefania De Pascalis, Silvia D’Ovidio, Stefania Crisci, Piera Maiolino, Rosaria De Filippi, Antonio Pinto

**Affiliations:** 1 Hematology-Oncology and Stem Cell Transplantation Unit, Istituto Nazionale Tumori, Fondazione ‘G. Pascale’, IRCCS, Via Mariano Semmola 49, 80131 Naples, Italy; 2 Department of Mental Health and Public Medicine, Section of Infectious Diseases, University of Campania Luigi Vanvitelli, Naples, Italy; 3 Pharmacy Unit, Istituto Nazionale Tumori, Fondazione ‘G. Pascale’, IRCCS, Naples, Italy; 4 Department of Clinical Medicine and Surgery, Università Degli Studi Federico II, Naples, Italy

**Keywords:** Multiple myeloma, Autologous stem cell transplantation, SARS-CoV-2 Omicron variant, Sotrovimab

## Abstract

**Background:**

To date, there is no information on the safety and efficacy of the novel anti-sarbecoviruses monoclonal antibody sotrovimab administered, as a post-exposure prophylactic measure, during the aplastic phase of autologous stem cell transplantation (ASCT).

**Methods:**

We describe the outcomes of a Multiple Myeloma (MM) patient, who was threateningly exposed to the Omicron (B.1.1.529) SARS-CoV-2 variant, two days after having received a myeloablative regimen of high-dose melphalan. The patient fulfilled all CDC criteria for prolonged close contacts with an index patient who tested positive for a molecular nasopharyngeal swab (Omicron; B.1.1.529) soon after admission to the ward. Given the high risks of morbidity and mortality in the case of COVID-19 developing during the aplastic phase of transplantation, we adopted a post-exposure prophylaxis intervention based on intravenous (i.v.) sotrovimab.

**Results:**

Sotrovimab (500 mg i.v.) was administered at day + 2 from stem cells reinfusion, *i.e.* 4 days after myeloablative chemotherapy, and at day + 5 from the last close contact with the Omicron-positive index case. The patient was fully protected from SARS-CoV-2 infection throughout his clinical course and remained molecularly negative at the day + 30 from the transplant. We compared times to engraftment and transplant-related toxicities of the sotrovimab-treated patient with the last 15 MM patients transplanted at our Centre, evidencing no unexpected safety signals, infusion-related reactions, or alarming effects on engraftment kinetics.

**Conclusions:**

We have shown here for the first time that administration of sotrovimab during the pre-engraftment phase of ASCT is effective, safe, and not associated with delays in hemopoietic recovery. As compared to MM patients who received the same myeloablative conditioning regimen, the patient given sotrovimab during the aplastic phase did not show any significant differences in engraftment kinetics and toxicity outcomes. Post-exposure prophylaxis with sotrovimab may represent a valuable approach in the stem cell transplantation setting for patients with high-risk exposure to a confirmed COVID-19 case sustained by highly infectious SARS-CoV-2 variants escaping the vaccine-derived immunity due to antigenic shifts in the spike proteins.

## Introduction

The multiple epidemic waves sustained by the severe acute respiratory syndrome coronavirus 2 (SARS-CoV-2) variants, including the lastly emerged Omicron (B.1.1.529) subtype, continue to pose unprecedented challenges for the management of patients with hemopoietic tumors. In this setting, the Omicron variant and its evolving sublineages represent a further significant threat. This is due to the enhanced transmissibility rate of these variants and their high potential for immune evasion in subjects who received a full course of mRNA SARS-CoV-2 vaccines [1].

Patients with Multiple Myeloma (MM), remain a highly vulnerable population in the context of the current phase of the SARS-CoV-2 pandemics [2]. This is linked to substantial disease- and treatment-related immunodeficiency but also to suboptimal responses to SARS-CoV-2 vaccines [2–4]. As compared to healthy individuals, MM patients, especially during or shortly after anti-myeloma treatments, display lower seroconversion rates after immunization with mRNA or viral vector vaccines along with delayed or inferior production of anti-SARS-CoV-2 neutralizing antibodies [4, 5]. These patients also show quantitative and functional impairments in specific T-cell responses to mRNA vaccines [6].

Thus, fully vaccinated MM patients maintain a significant risk of breakthrough infections possibly leading to the enhanced likeliness of severe clinical outcomes [2, 3]. A survey of 1182 vaccinated MM patients showed a fivefold higher risk of breakthrough SARS-CoV-2 infections as compared with matched patients with non-malignant conditions and an eightfold increase in hospitalization risk [7]. These threats may markedly increase if MM patients contract the infection throughout stem cell transplantation (SCT), a procedure itself associated with a profound and prolonged treatment-related immunosuppression [8]. In addition, recipients of SCTs who develop COVID-19 are at significant risk of morbidity and mortality [9]. An aggressive policy of surveillance and infection prevention is therefore mandated for these patients [2, 8, 9].

Here we first report on the safety and efficacy of sotrovimab (VIR-7831/GSK4182136), a novel neutralizing anti-sarbecoviruses monoclonal antibody (mAb), administered as post-exposure prophylaxis during the pre-engraftment phase of autologous SCT (ASCT) to a MM patient threateningly exposed to the Omicron (B.1.1.529) SARS-CoV-2 variant [10].

## Methods

The study was conducted according to the guidelines of the Declaration of Helsinki and its later amendments. Approval by the Institutional Review Board was not needed given the nature of the study. The patient signed a written informed consent to off-label treatment with sotrovimab and a further written consent allowing the publication of the present report. All transplanted patients signed a written informed consent (EBMT-F-023-01) allowing the use of anonymized registered clinical data for scientific purposes.

Patients with MM (n = 15) received induction treatment with bortezomib, thalidomide and dexamethasone (VTD), except one case who was given bortezomib, cyclophosphamide, and dexamethasone (VCD). In addition, two patients also received a second-line therapy based on carfilzomib and desamethasone before the transplant. Peripheral stem cell mobilization was achieved with steady-state granulocyte colony-stimulating factor (filgrastim) in 10 patients, single-agent cyclophosphamide (3.0 g/m^2^) in 2 cases and a combination of vinorelbine (25 mg/m^2^) and cyclophosphamide (1500 mg/m2) in 3 patients. Standard high-dose chemotherapy (HDT) with single-agent melphalan (200 mg/m^2^) was adopted as a conditioning regimen before ASCT in all patients, as well as subcutaneous peg-filgrastim (6 mg) given at day + 4 after stem cells reinfusion.

For sequencing of viral isolates, RNA was extracted from nasopharyngeal swabs (NPS) using QIAamp Viral RNA Mini Kit (Qiagen, Hilden, Germany) according to the manufacturer's protocol. Viral genomes were amplified by performing a multiplex approach, using version 1 of the CleanPlex SARS-CoV-2 Research and Surveillance Panel (Paragon Genomics, Hayward, United States), following the manufacturer’s protocol starting with 50 ng of total RNA and followed by Illumina sequencing on a NextSeq 500 (Illumina, San Diego, United States). Libraries were controlled with a High Sensitivity Labchip and quantified with Qubit Fluorometric Quantitation system (Thermo Fisher Scientific, Waltham, United States). Raw data were trimmed and analyzed by popular bioinformatics software CLC workbench 5, and Basic Local Alignment Search Tool (BLAST).

## Results

On December 27, 2021, two patients were admitted to our ward for high-dose chemotherapy (HDT) and ASCT. They shared a two-bed positive-pressure isolation room with high-efficiency particulate air filters and both had a negative molecular NPS the day before admission.

Patient 1 (the index case), was a 31-year-old male with relapsed Hodgkin lymphoma. He was not yet vaccinated due to former COVID-19 pneumonia, sustained by the SARS-CoV-2 VUI202012/01 GRY alpha variant (B.1.1.7), which occurred one year before (December 2020). He had recovered from pneumonia after 25 days with negative serial NPSs starting from 35 days after the first COVID-19 diagnosis. Pre-transplant salvage chemotherapy was administered without problems and the patient displayed a sequence of negative NPSs throughout treatment.

Patient 2, was a 61-year-old male with MM who had received two doses of the BNT162b2 mRNA vaccine on October 22 and November 12, 2021. He was given induction with bortezomib, thalidomide, cyclophosphamide and dexamethasone, which resulted in a stringent complete remission. He was a poor mobilizer with a total stem cell harvest of 2 × 10^6^ CD34 + /Kg.

The case index was planned to initiate HDT on January 3, 2022, while the MM patient received HDT (melphalan 200 mg/m^2^; 400 mg total dose) on December 31, 2021 (day-2). Since per internal procedures we screen all inpatients for SARS-CoV-2-RNA every 3–4 days, both patients underwent a further NPS on December 30, 2021. Results, forwarded on December 31, evidenced that while the MM case (patient 2) had a negative NPS, the case index (patient 1) tested positive. Upon further questioning, the patient disclosed that, in contrast with the information provided at admission, he had unprotected contact at home with his brother. The latter was found positive at a molecular NPS performed on December 29, 2021, due to unremitting fever, headache, and pharyngodynia.

The index case (patient 1) not having yet started HDT was moved to an academic infectious diseases Unit, for sequencing of the viral isolate and anti-viral treatment. The analysis of the SARS-CoV-2 RNA isolated from his NPS showed the presence of VOC-21NOV-01 (B.1.1.529) viral variant, named Omicron variant. Therefore, the patient was treated with intravenous sotrovimab (500 mg flat dose) on day + 3 from infection and with remdesivir on days + 3–5 (200 mg on the first day and 100 mg on the second and third day). On day + 9, the index case achieved a negative NPS and was discharged.

Unfortunately, the MM case (patient 2) fulfilled all the CDC close contact criteria with the index patient (patient 1) with whom he had shared a positive air pressure room for four days and had several risk factors for severe COVID-19 [4–6, 8, 9]. In addition, despite two doses of BNT162b2 mRNA vaccine, the patients lacked neutralizing antibodies [4]. Based on these considerations, we decided to administer post-exposure prophylaxis with sotrovimab despite the patient having already received ASCT and was in the pre-engraftment phase. We opted for sotrovimab due to its capacity, at variance with antibody cocktails, such as casirivimab and imdevimab or etesevimab and bamlanivimab, to recognize a viral epitope not substantially altered by the mutations typically found in the Omicron spike proteins [1, 10–13].

Therefore, on January 5, 2022 (day + 2 from ASCT and day + 5 from the last close contact with the Omicron-positive index case), the patient, after signing informed consent, was given intravenous sotrovimab (500 mg). At the time of infusion, his hemogram showed an absolute neutrophil count (ANC) of 4.9 × 10^9^/L, an absolute lymphocyte count (ALC) of 0.3 × 10^9^/L, platelets 216 × 10^9^/L, and hemoglobin of 12.7 g/dL. From December 29, 2021 (day-5), he was given prophylactic levofloxacin, fluconazole, and acyclovir plus sinusoidal obstruction syndrome prevention with heparin (100 IU/kg/day as a 24-h infusion). On January 7 (day + 4) the patient received subcutaneous peg-filgrastim (6 mg). Two irradiated platelet units were infused on January 11 and 12 (days + 8 and + 9) while he never necessitated RBC support. His clinical course was uneventful, without febrile episodes or hepatic and renal toxicities; mucositis and nausea/vomiting were never higher than grade 1. Daily NPSs were negative up to discharge and engraftment times were as follows: ANC > 500 and > 1000 × 10^9^/L (10 and 11 days, respectively), platelets > 20.000 and > 50.000 × 10^9^/L (11 and 13 days, respectively), and ALC > 1000 × 10^9^/L (12 days). Repeated post-discharge bi-weekly NPSs were constantly negative and symptoms possibly related to SARS-CoV-2 infection never manifested up to the last follow up (day + 30) post-transplant.

**Fig. 1:**
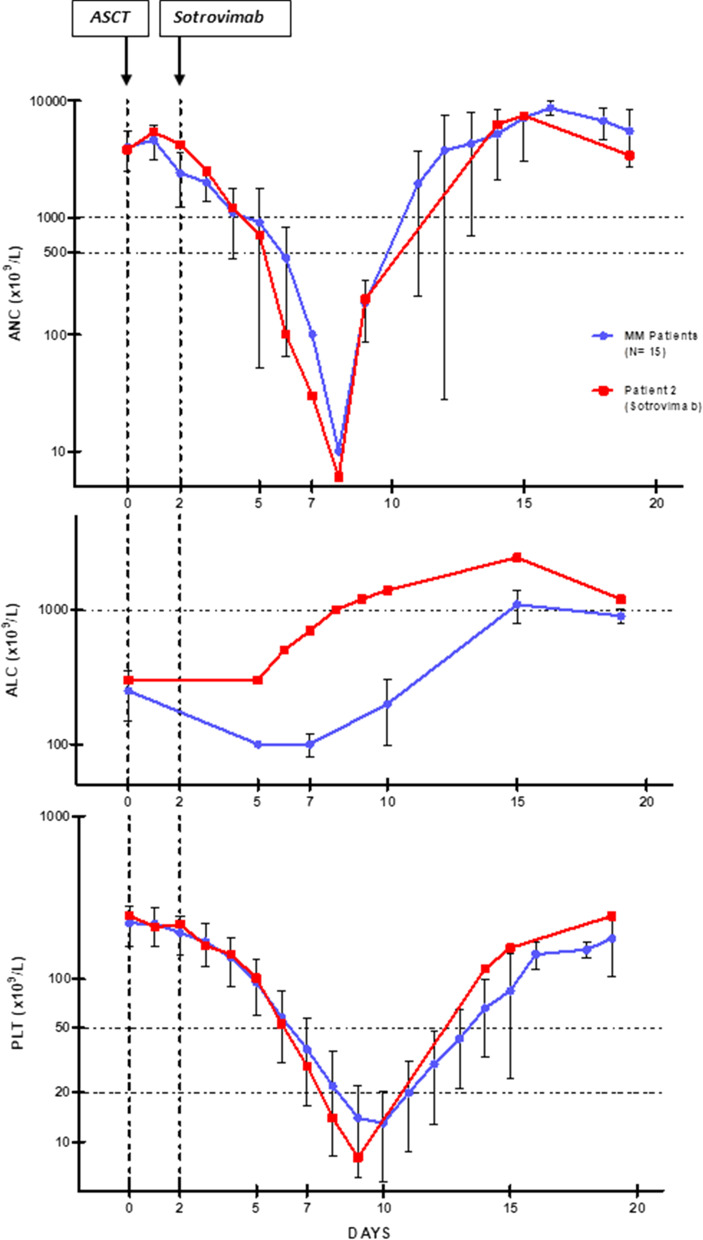
Visual clinical timeline including treatments and engraftment kinetics of a MM patient (patient 2) treated with sotrovimab during the pre-engraftment phase of autologous stem cell transplant (ASCT) in comparison with data from the last 15 MM patients who underwent the same procedure at our institution in the preceding four months. Blue lines represent median values (± SD) for absolute neutrophil count (ANC), absolute lymphocyte count (ALC) and platelets counts obtained from the last 15 MM patients who received high-dose melphalan (200 mg/m^2^) and autologous stem cell transplant (ASCT) before patient 2 at our Center. The red lines indicate ANC, ALC and platelet counts for a MM patient (Patient 2) given high-dose melphalan (200 mg/m^2^) and single-agent sotrovimab (500 mg flat dose), two days after ASCT

The case timeline as compared to the last 15 MM patients who received ASCT with the same conditioning at our Center is illustrated in Fig. 1. Clinical and transplant-related features of these patients were fully comparable with those of the sotrovimab-treated patient, including the total dose of melphalan receveid during high-dose conditioning and the total dose of CD34+ cells reinfused (Table 1). As shown in Fig. 1, no significant differences in engraftment kinetics emerged as a consequence of sotrovimab administration. Nadir peaks for ANC, platelets, and ALC were fully comparable (Fig. 1), as well as, the median times to engraftment for ANC > 500 and > 1000 × 10^9^/L (10 ± 1 days for both outcomes), platelets > 20.000 and > 50.000 × 10^9^/L (12 ± 1 and 13 ± 2 days, respectively) and ALC > 1000 × 10^9^/L (18 ± 4 days). Similarly, the incidence and grading of transplant-related toxicities and the required hemocomponents support were superimposable (Table 1).

**Table 1:** Clinical characteristics of MM patients

	N° (%)
	(N = 15)
Age at transplant, (years)	
Median (range)	58 (43–70)
Gender	
Male	9 (60)
Female	6 (40)
Paraprotein isotype	
IgG/κ	7 (47)
IgG/λ	3 (20)
IgA/κ	1(7)
Light chain/κ	2 (13)
Light chain/λ	1 (7)
Nonsecretory	1 (7)
Frontline MM therapy	
VTD	14 (93)
VCD	1 (7)
Stem Cells Mobilization procedure	
G-CSF steady-state	10 (67)
Vinorelbine-Cyclophosphamide	2 (13)
High-dose Cyclophosphamide	3 (20)
Total CD34 + cells yield (× 10^6^/kg)	
Median (range)	4.54 (2.6–8.4)
High-dose conditioning (Melphalan 200 mg/m^2^)	
Total mg.s of Mel delivered (median, range)	376 (278–430)
Total CD34 + cells infused (× 10^6^/kg)	
Median (range)	2.9 (2.4–4.7)
Hemogram at day 0 [median (range)]	
ANC × 10^9^/L	4.0 (3.7–6.4)
Platelets × 10^9^/L	220 (123–310)
ALC × 10^9^/L	0.2 (0.0–0.5)
Hemocomponents support up to discharge	
RBC units transfused (median, range)	0 (0–3)
Platelet units transfused (median, range)	1 (0–3)
Transplant-related Toxicity (G1-G2)^*^	
Febrile neutropenia	2 (13)
Fever of unknown origin	3 (20)
Microbiologically documented infection	2 (13)
Mucositis	7 (47)
Diarrhoea	6 (40)
Other adverse events	1 (7)

^*^N° of patients experiencing G1-G2 events. Toxicity was assessed using Common Terminology Criteria for Adverse Events version 4.0. MM, multiple myeloma; VTD, bortezomib, thalidomide, dexamethasone; VCD, bortezomib, cyclophosphamide, dexamethasone

## Discussion

Our results indicate that post-exposure prophylaxis with sotrovimab shortly after HDT/ASCT, and before full engraftment, is feasible, safe, and effective. No unexpected safety signals, including infusion-related reactions, or alarming effects on engraftment kinetics emerged. Namely, administration of sotrovimab to our patient did not alter the toxicity profile of the ASCT procedure nor caused any delay in hematologic recovery times or increased need for supportive treatments, as compared to the standard outcomes of other MM patients who received the same type of transplantation.

These findings add to the reported safety profile for sotrovimab taking into account that the antibody was infused shortly after administration of a myeloablative regimen. Since early pharmacokinetic data from the COMET-ICE trial projected a terminal elimination half-life of about 32 days for sotrovimab, it is conceivable that bioactive concentrations of the antibody were present in our patient throughout the aplastic phase up to transplant engraftment [10, 14]. This suggests that sotrovimab is devoid of significant toxicity toward human hemopoietic CD34+ precursors and/or their early progeny.

As shown by our report even stringent measures are unable to completely prevent SARS-CoV-2 transmission within SCT units, especially during outbreaks of highly infectious viral variants such as Omicron [15]. Since the positive air pressure environment may contribute to viral spread and transplant recipients present multiple disease- and treatment-related risk factors for severe COVID-19, aggressive pre-emptive strategies are needed in such a clinical setting [2, 8, 9].

Post-exposure prophylaxis with mAbs may then represent a possible option to prevent SARS-CoV-2 infection or mitigate the development of severe COVID-19 in high-risk patients such as those affected by MM under active treatment and those receiving SCTs. Studies during the first phase of SARS-CoV-2 pandemics indicated mortality rates up to 20% for COVID-19 developing in these patients [2]. In MM patients who received SCTs, reported mortality rates at 30 days after COVID-19 diagnosis were 32% and 33% for allo-SCT and ASCT recipients, respectively [9]. Due to their suboptimal humoral and cellular responses to SARS-Cov-2 vaccines, these patients remain at a higher risk of breakthrough infections during the current Omicron epidemics [4–7].

We deemed then appropriate to offer post-exposure prophylaxis in this specific case due to the high transmission rate of the Omicron variant, conceivably enhanced by the positive pressure environment, the deep lymphocyte nadir expected with high-dose melphalan, and the possible delay in hemopoietic reconstitution given the relatively low number of CD34+ progenitors infused.

Sotrovimab is pan-sarbecovirus IgG1 mAb first tested for early prevention of Covid-19 progression in high-risk patients. In a multicenter randomized trial, for non-hospitalized patients with symptomatic Covid-19 and at least one risk factor for disease progression, administration of a single 500 mg infusion of sotrovimab led to a statistically significant reduction (85%) of the relative risk of hospitalization or death vs. placebo (*P* = 0.002) [10]. Given the ability of sotrovimab to lower the risk of disease progression and its unaltered potency to recognize and neutralize the recently emerged SARS-CoV-2 Omicron variant, we identified this mAb as an optimal candidate for post-exposure prophylaxis in our MM patient [10–13].

Within the limits of a single case report, our results suggest that sotrovimab is a safe and effective tool to avoid deleterious delays in ASCT for high-risk patients threateningly exposed to present and emerging highly infectious SARS-CoV-2 variants escaping the vaccine-derived immunity due to antigenic shifts in the spike proteins [11–13]. Post-exposure prophylaxis may turn of special relevance in the setting of SCTs for highly immunocompromised patients ineligible for SARS-CoV-2 vaccination due to medical contraindications and those unable to mount an adequate immune response after vaccination.

## Data Availability

The datasets generated during and/or analysed during the current study are available from the corresponding author on a reasonable request.
